# Feto-Maternal Crosstalk in the Development of the Circadian Clock System

**DOI:** 10.3389/fnins.2020.631687

**Published:** 2021-01-12

**Authors:** Mariana Astiz, Henrik Oster

**Affiliations:** Center of Brain, Behavior and Metabolism, Institute of Neurobiology, University of Lübeck, Lübeck, Germany

**Keywords:** pregnancy, fetus, circadian clock, placenta, gating

## Abstract

The circadian (24 h) clock system adapts physiology and behavior to daily recurring changes in the environment. Compared to the extensive knowledge assembled over the last decades on the circadian system in adults, its regulation and function during development is still largely obscure. It has been shown that environmental factors, such as stress or alterations in photoperiod, disrupt maternal neuroendocrine homeostasis and program the offspring’s circadian function. However, the process of circadian differentiation cannot be fully dependent on maternal rhythms alone, since circadian rhythms in offspring from mothers lacking a functional clock (due to SCN lesioning or genetic clock deletion) develop normally. This mini-review focuses on recent findings suggesting that the embryo/fetal molecular clock machinery is present and functional in several tissues early during gestation. It is entrained by maternal rhythmic signals crossing the placenta while itself controlling responsiveness to such external factors to certain times of the day. The elucidation of the molecular mechanisms through which maternal, placental and embryo/fetal clocks interact with each other, sense, integrate and coordinate signals from the early life environment is improving our understanding of how the circadian system emerges during development and how it affects physiological resilience against external perturbations during this critical time period.

## Introduction

The circadian system is required to anticipate and adapt physiology to daily recurring changes in the environment over 24 h (Dibner et al., 2010). It coordinates complex behaviors such as sleep (Collins et al., 2020), activity (Moore and Eichler, 1972; Stephan and Zucker, 1972), food intake (Hatori et al., 2012; Koch et al., 2020), and stress responses (Oster et al., 2006). In mammals, a master circadian pacemaker is located in the hypothalamic suprachiasmatic nucleus (SCN) and subordinated clocks are present throughout the brain and the periphery (Ralph et al., 1990). The SCN perceives time of day via direct photic input from the retina and subsequently relays temporal information through coordination of the neuroendocrine system. Therefore, several SCN efferent connections are found within the medial hypothalamus where key cell groups are involved in organizing hormone release and autonomic nervous system tone (Kalsbeek et al., 2006, 2011). A plethora of humoral and neuronal signals convey time-of-day information to the periphery to elicit rhythmic regulation of the local clock gene machinery and, in turn, of a set of tissue-specific downstream clock-controlled genes (Buhr and Takahashi, 2013).

During pregnancy, the maternal neuroendocrine system adapts to support fetal development and growth (Tal et al., 2000; Russell and Brunton, 2019). Circadian coordination likely plays a fundamental role in this adaptation during the whole period of pregnancy, parturition, and lactation (Wharfe et al., 2016a). However, compared to the extensive knowledge gained over the last decades on the adult circadian system, its regulation and function during pregnancy remains largely obscure (Wharfe et al., 2011, 2016a,b; Papacleovoulou et al., 2017). The placenta is the only organ that is formed by the interaction of, both, maternal and fetal/embryonic tissues. It forms the interface between the two circulatory systems. The circadian clock is strongly involved in regulating functions such as hormone synthesis and immunity in the adult, then it might be involved in the diurnal regulation of these functions also during embryogenesis and in the placenta. And, last but not least, the fetal/embryo circadian system develops and gains autonomy toward term (Wharfe et al., 2011; Landgraf et al., 2015) under the influence of endogenous and exogenous entrainment signals crossing the placenta (Serón-Ferré et al., 2012; Čečmanová et al., 2019). However, little is known about fetal/neonate clock functions that might be relevant during this period of development.

**FIGURE 1:**
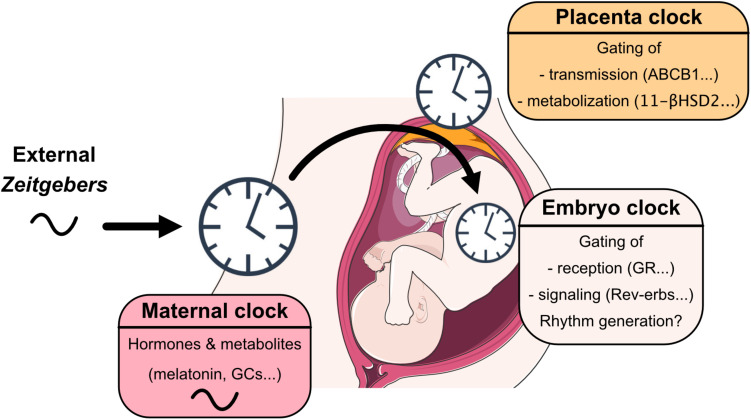
View on mechanisms through which maternal, placental and embryo/fetal clocks interact with each other, sense, integrate, and coordinate signals from the early life environment.

Understanding circadian coordination during pregnancy requires an assessment of the interaction of three clocks—maternal, placental and fetal—plus taking into account that this interaction undergoes dynamic changes over the course of pregnancy (Mark et al., 2017). After birth, maternal behavior, body temperature and signals from breast milk further affect neonate circadian system development until weaning (Nozhenko et al., 2015) (Figure 1).

### The Maternal Circadian System During Pregnancy and Early Postnatal Life

The role of the maternal clock during perinatal life has been studied by SCN lesion experiments in rodents, using clock deficient models and by exposing the pregnant mother to environmental conditions such as constant light (LL), chronic phase shifts or mistimed food availability, at different phases of gestation (Varcoe et al., 2011, 2013, 2018; Vilches et al., 2014; Houdek et al., 2015; Mendez et al., 2016; Smarr et al., 2017; Carmona et al., 2019). In the short term, the impact of maternal chonodisruption has been assessed using within-litter synchrony of the fetal/neonate central and peripheral clocks, metabolic rhythms, and activity as readouts. Interestingly, all different manipulations seems to have similar effects depending on the time of gestation when the chonodisruption was induced (Reppert and Schwartz, 1984; Davis and Gorski, 1988; Jud and Albrecht, 2006; Mendez et al., 2012, 2016; Varcoe et al., 2016; Salazar et al., 2018). Maternal chronodisruption also induces long term effects in the offspring such as memory and learning deficits (Vilches et al., 2014), increased anxiety, anhedonia, and depressive-like behavior (Voiculescu et al., 2016; Zhang et al., 2017) and metabolic effects such as adiposity and impaired glucose tolerance (Mendez et al., 2016).

Several maternal signals have been proposed as candidates to cross the placenta and reach the fetal clock. Melatonin is secreted by the pineal gland at night controlled through neuronal connections from the SCN (Lehman et al., 1987). Melatonin levels increase gradually toward the end of pregnancy returning to non-pregnant levels shortly after birth (Tamura et al., 2008). Melatonin is also found at considerable amounts in breast milk (Illnerová et al., 1993; Rowe and Kennaway, 2002). Experiments in rats have demonstrated that some of the short- and long-term effects of maternal LL exposure (Mendez et al., 2012; Houdek et al., 2015; Voiculescu et al., 2015, 2016), pinealectomy (Bellavía et al., 2006; Motta-Teixeira et al., 2018) or SCN lesions (Davis and Mannion, 1988) can be rescued by the administration of melatonin (recently reviewed by Hsu and Tain, 2020). Interestingly, the central and peripheral clocks of the fetus/newborn seem to respond differently to melatonin replacement in arrhythmic mothers (Mendez et al., 2012; Houdek et al., 2015). Despite that melatonin receptors have been found in several fetal tissues and in different species (Torres-Farfan et al., 2006) as previously reviewed by Voiculescu et al. (2014), the melatonin secretion pathway is suppressed in most inbred mouse strains. Considering that the offspring of these mice shows robust rhythms, melatonin might be a synchronizing signal for the fetal/neonate clock, but it is likely not essential for the normal development of the circadian system.

Dopamine has been proposed as a “light-phase” entrainment signal—i.e., antiphasic and functionally antagonistic to melatonin—during the development of the circadian system (Iuvone and Gan, 1995). Dopamine is able to cross placental barrier freely and is also found in breast milk (Watanabe et al., 1990). Moreover, dopamine receptors are widely expressed in the fetal/neonate brain (Weaver et al., 1992; Rivkees and Lachowicz, 1997). The exposure of neonates to the dopamine receptor 1 (D1R) agonist SKF38393 increases c-fos expression in the SCN (Weaver and Reppert, 1995). However, there is no substantial evidence of a direct role of dopamine programing the long-term function of the circadian system.

Glucocorticoids (GCs) have strong circadian entrainment functions (Oster et al., 2017). In humans and rodents, maternal GCs are released rhythmically anticipating the active phase during whole pregnancy with a gradual increase of baseline levels toward the end (Wharfe et al., 2016a). GCs are essential for fetal tissue maturation, especially in the lung, and GR (glucocorticoid receptor), or CRH (corticotrophin releasing hormone) deficiency is lethal for the fetus (Goldfeld et al., 1983; Muglia et al., 1995). Therefore, while low GC concentrations seem to be necessary for pregnancy success, epidemiological studies and animal experiments suggest that high GC levels during pregnancy increase the risk of developing behavioral and metabolic disorders later in life (Moisiadis and Matthews, 2014a; Coleman et al., 2016; Marín, 2016; Busada and Cidlowski, 2017; Van den Bergh et al., 2017; Logan and McClung, 2019). Interestingly, most rodent prenatal stress paradigms entail some degree of circadian disruption because the animals are manipulated during their normal rest phase. We have recently demonstrated that the offspring from mothers exposed to GCs during the rest phase show worse circadian and stress-related behavioral phenotypes than those from mothers exposed to the same GC concentration, but during the active phase (Astiz et al., 2020).

Much less is known about other signals that are also rhythmic in the mother and are known to cross the placenta or to impact on fetus development such as leptin. Transplacental transport of leptin increases during the last week of gestation in rats, together with an increase in expression of leptin receptor in the placenta, likely due to increasing energy requirements (Herrid et al., 2014; Vlahos et al., 2020). Interestingly, transplacental leptin passage is reduced after maternal GCs exposure, whereas treatment with metyrapone (an inhibitor of GCs synthesis) has the opposite effect (Smith and Waddell, 2002, 2003). Other signals such as placental lactogen, prolactin, progesterone, estradiol, and insulin are less likely candidates for fetal circadian entrainment. Serum levels of human chorionic gonadotropin (hCG) and placental lactogen (hPL) were measured over 24 h, but no clear rhythms were detected (Houghton et al., 1982). Progesterone, estradiol, and insulin show rhythmic oscillations in non-pregnant rodents but there is not enough evidence for such rhythms during pregnancy.

Circadian rhythms in maternal core body temperature were also investigated as a possible entrainment signal, however, the reduced amplitude of these rhythms argues against a significant role as time-giver (Wharfe et al., 2016b).

Taking together these data suggest that the effect of maternal signals on the developing circadian system depend on concentration, circadian phase, the interaction with other signals, and gestational/postnatal age.

### Placental Clocks and the Circadian Regulation of Feto-Maternal Crosstalk

In order to reach the developing embryonic/fetal clock, entrainment signals will have to pass through the placenta—as process, which could be by itself gated by the circadian clock. The placenta provides the interface between both circulatory systems. It controls the exchange of nutrients, hormones, xenobiotics, metabolites, and waste between mother and fetus (Han et al., 2018; Staud and Karahoda, 2018). Some maternal signals such as melatonin or dopamine freely cross the placenta and convey external time to the fetus (Naitoh et al., 1998; Okatani et al., 1998). Others, such as glucocorticoids, are metabolized by enzymes expressed in the labyrinth zone (LZ) of the placenta (Okatani et al., 1998; Krozowski et al., 1999; Mark et al., 2009, 2017; Christ et al., 2012; Waddell et al., 2012; Houdek et al., 2015). The LZ of rodents consists of maternal blood spaces separated from the fetal vasculature by trophoblasts and fetal connective tissue. It is of fetal origin and analog to the chorionic villi in humans (Han et al., 2018; Staud and Karahoda, 2018). Enzymes such as 11-βHSD2 (11-β-hydroxysteroid dehydrogenase 2) and ABCB1 (ATP-Binding Cassette Subfamily B Member 1) are highly abundant in the LZ and protect the fetus from excessive levels of GCs. The expression of these enzymes is rhythmic in the circadian range in the LZ and other tissues (Waddell et al., 2012). For instance, ABCB1 has drug-efflux functions in placenta with a broad substrate specificity, a diurnal regulation might have implications when considering the optimal treatment time of pregnant mothers aiming at either maximal or minimal availability to the fetus. Therefore, it would be interesting to assess whether the local clock is responsible for the rhythmic regulation of these or other transporters.

In mice, the junctional zone (JZ) of the placenta secretes monoamines and steroids with endocrine, paracrine, and autocrine functions modulating maternal and fetal physiology throughout pregnancy (Longhi and Kulay, 1974; Napso et al., 2018). Placental hormones such as hCG (human chorionic gonadotropin), hPL (placental lactogen) show no significant diurnal variation in maternal serum (Houghton et al., 1982) which is probably explained by the absence of a robust rhythmic expression of the clock gene machinery in the JZ (Wharfe et al., 2011). The placental decidua mediates the maternal immune tolerance to the embryo (Arck and Hecher, 2013). Since, several immune processes are strongly regulated by the circadian system, it would be interesting to assess whether either the maternal or the placental clock influence this aspect of immune adaptation.

### Fetal and Neonate Clock Development and Their Function as Gatekeepers of Circadian Entrainment Signals

Besides maternal signals and their passage through the placenta, the entrainment of the fetal circadian system will depend on a third factor, the reception of those signals at embryonic target tissues. The expression of receptors for dopamine and glucocorticoids shows dynamic changes in the developing SCN with high levels during the prenatal phase followed by downregulation during postnatal stages (Rosenfeld et al., 1988; Weaver and Reppert, 1995). The exposure of neonates to the dopamine receptor 1 (D1R) agonist SKF38393 increases c-fos expression in the SCN during the first 3 days of postnatal life, but receptor expression is downregulated by post-natal day 4—and so is the response of the SCN to the D1R agonist (Weaver and Reppert, 1995). GCs influence the development of many hypothalamic nuclei including the SCN (Moisiadis and Matthews, 2014b; Čečmanová et al., 2019) but the GR is not expressed in the adult nuclei (Rosenfeld et al., 1988). Consequently, the adult master clock becomes insensitive to dopamine, GCs and, potentially, other peripheral signals, which may be an essential condition for the SCN to keep the time under conditions of conflicting environmental signals.

When exactly and how the circadian clock starts ticking is still an open question. In mice, neuronal division in the developing SCN takes place between gestational day (GD) 10–15 peaking at GD12 (Kabrita and Davis, 2008). Intra-SCN circuits differentiate during the following days and retinal projections reach the SCN mediating photic entrainment shortly after birth (Sekaran et al., 2005). In contrast, the molecular clock machinery in the SCN and peripheral tissues is already expressed earlier (Landgraf et al., 2015; Čečmanová et al., 2019) and daily changes in metabolic activity are detectable in the SCN during late gestation (Reppert and Schwartz, 1984). Recent data from our lab show that the circadian phase of GCs that reach fetal tissues determines their effectiveness in programing the offspring’s circadian behavior. This temporal gating originates from the embryonic clock system and may involve rhythmic expression of the negative GR modulator *Reverse erythroblastoma (REV-ERB*α/β aka *Nr1d1/2*) (Astiz et al., 2020).

Taken together, these results indicate that an intrinsic genetic programs, at the level of fetal tissues, interact with maternal signals. The outcome of this interaction not only affects acute responses of the embryo to external stimuli, but may also determine the physiological programing of circadian behavior and energy metabolism.

## Discussion

Circadian clocks have a pervasive influence on all aspects of physiology and behavior and, not surprisingly, they also influence embryonic development and the interaction between the embryo and its prenatal environment. Potent players in this context are timing signals perceived by the mother and transmitted to the unborn. On the other hand, the fetal circadian system is gradually evolving toward the end of gestation, thus more and more impinging on how maternal signals are interpreted and translated. Placental rhythmic programs have an important function in this crosstalk by gating which signals actually reach the embryo and how much of them at a given time.

The downregulation of receptors (such as GR) in the fetal SCN and the partial loss of rhythmicity of some of the maternal signals toward term indicate an emancipatory step of the prenatal pacemaker from maternal zeitgebers. It also highlights dynamics in the interaction between maternal signals and developmental programs during pregnancy in general. Metabolomic approaches may help to further decipher these kinetics allowing more straightforward strategies to manipulate genes and pathways during different stages of fetal development. From a clinical perspective, a better comprehension of these interactions will allow to improve existing therapeutic paradigms targeting disorders of the pregnant mother or the developing child with regard to efficiency or unwanted side effects.

## Author Contributions

MA and HO discussed the concept and wrote the manuscript. Both authors contributed to the article and approved the submitted version.

## Conflict of Interest

The authors declare that the research was conducted in the absence of any commercial or financial relationships that could be construed as a potential conflict of interest.
